# Pancreatic Panniculitis: A Case Associated With Acute Pancreatic Allograft Rejection

**DOI:** 10.7759/cureus.52925

**Published:** 2024-01-25

**Authors:** Avraham Kohanzadeh, Ariel Sher, Olivia Wind, Qiang Liu, Pooja Srivastava, Bijal Amin, Ranon Mann

**Affiliations:** 1 Internal Medicine, Albert Einstein College of Medicine, New York City, USA; 2 Dermatology, New York Medical College, New York City, USA; 3 Dermatology, Mount Sinai Hospital, New York City, USA; 4 Pathology and Laboratory Medicine, Montefiore Medical Center, New York City, USA; 5 Dermatopathology, Montefiore Medical Center, New York City, USA; 6 Dermatology, Montefiore Medical Center, New York City, USA

**Keywords:** allograft rejection, transplant rejection, dermatology, pancreas transplant, pancreatic panniculitis

## Abstract

We present a unique case of pancreatic panniculitis (PP) in a 42-year-old male with a history of pancreas-after-kidney (PAK) transplant. The patient developed PP due to acute pancreas allograft rejection. Clinical manifestations included fevers, myalgias, arthralgias, and tender erythematous subcutaneous nodules on the lower extremities. A recent hospital admission was noted for acute pancreas allograft rejection related to low tacrolimus levels. Rheumatological and infectious disease workups were negative. Skin nodule punch biopsy confirmed PP with lobular panniculitis, necrotic adipocytes, basophilic debris, and calcification. Pancreatic biopsy showed evidence of parenchymal acute cellular rejection. Lipase and amylase levels were elevated (1781 U/L and 881 U/L, respectively). Treatment involved pulse solumedrol and thymoglobulin for pancreatic rejection, alongside adjustments to immunosuppressive medications. This case highlights the rarity of PP in a PAK recipient and its association with acute pancreas allograft rejection. Importantly, it is the first reported case of PP occurring solely in the context of pancreas transplant rejection, without concurrent kidney damage or rejection. Prompt diagnosis and management led to the resolution of skin and systemic symptoms. In conclusion, this report presents a clinically relevant and unique case of PP resulting from acute pancreas allograft rejection in a PAK transplant recipient. The findings underscore the importance of early diagnosis and management for positive patient outcomes, serving as a reminder to consider underlying pancreatic pathology when encountering PP in transplant recipients.

## Introduction

Pancreatic panniculitis (PP) is a rare inflammatory dermatological condition, occurring in 2-3% of patients with pancreatic disease. Acute pancreatitis is the most common pancreatic disease to present with PP, followed by pancreatic cancer and pancreatic pseudocysts [1-3]. PP has also been described in kidney transplant recipients secondary to native pancreatitis [4-6]. However, PP arising secondary to pancreatic allograft rejection is uncommon and has rarely been reported in the literature [5-11].

PP causes tender and erythematous subcutaneous nodules affecting the distal lower extremities and, less commonly, the trunk. Nodules can ulcerate and drain an oily brown substance caused by coagulative necrosis of adipocytes. These are characteristic findings that distinguish PP from other dermatological conditions, such as other forms of panniculitis, vasculitis, and infection [2].

PP is associated with elevated levels of lipase, amylase, phosphorylase, and trypsin [2]. Enzymes circulate and deposit in tissues, triggering polyarthritis, myalgias, and panniculitis. The enzymatic activity of lipase within the subcutaneous tissues leads to the breakdown of local triacyl-glyceride polymers, with subsequent liberation of glycerol and free fatty acids (FFA) monomers. The combined effects of enzymatic lipase activity and monomer buildup within the subcutaneous tissues lead to dystrophic calcification and the formation of tender erythematous skin nodules. The typical histopathological findings of PP display neutrophils and necrosis of lobular adipose tissue, commonly referred to as "ghost cells" [2, 12-15]. A diagnosis of PP, established histologically, necessitates thorough evaluation for pancreatic disease, as treatment targets the underlying cause.

The prognosis of PP is dependent on the underlying trigger. When PP develops in patients with pancreatitis, the nodules typically resolve after pancreatic inflammation subsides. In patients with underlying pancreatic carcinoma, the presence of PP is more challenging to treat [15]. It is imperative to screen for underlying pancreatic disease in patients who present with PP. In cases where there appears to be no underlying pancreatic disease, the presence of PP can be indicative of early-stage pancreatic cancer.

## Case presentation

**Figure 1:**
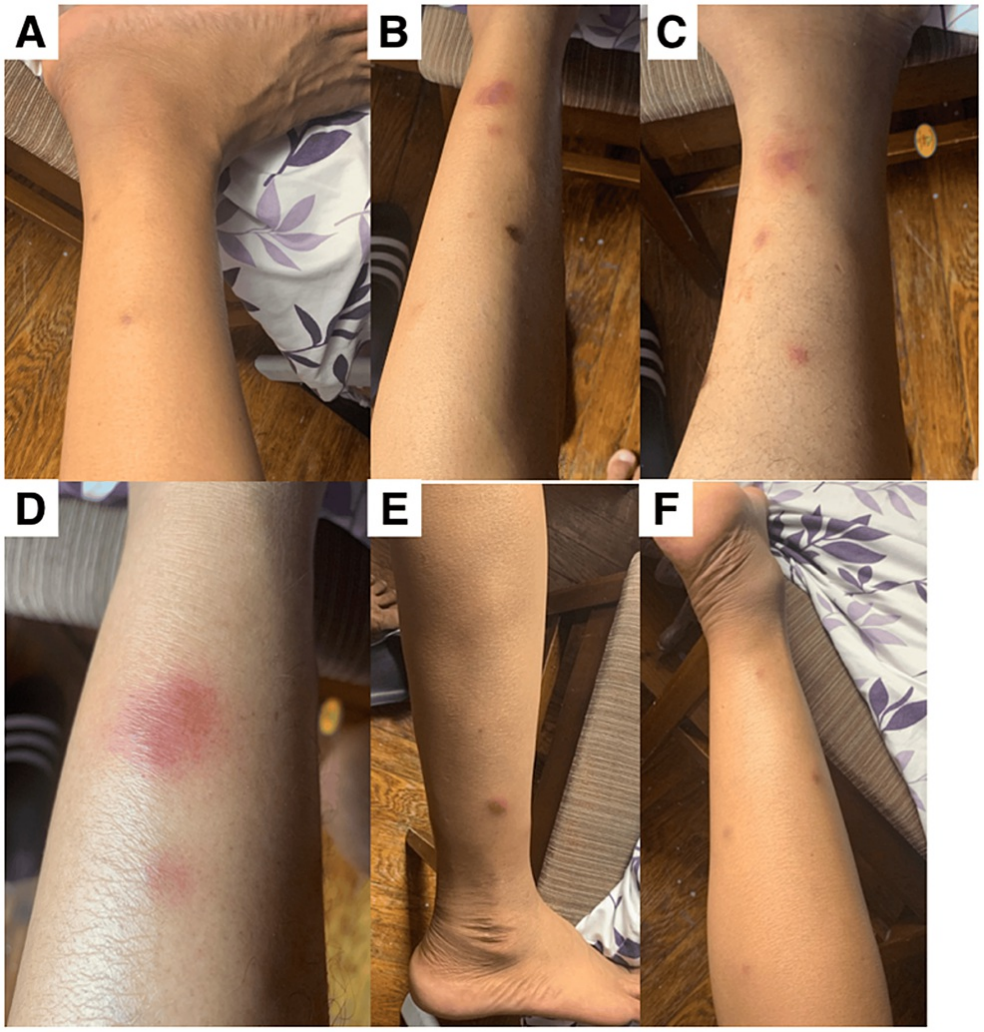
Pancreatic panniculitis - example of numerous tender, erythematous, and violaceous subcutaneous nodules on the bilateral lower extremities

**Figure 2:**
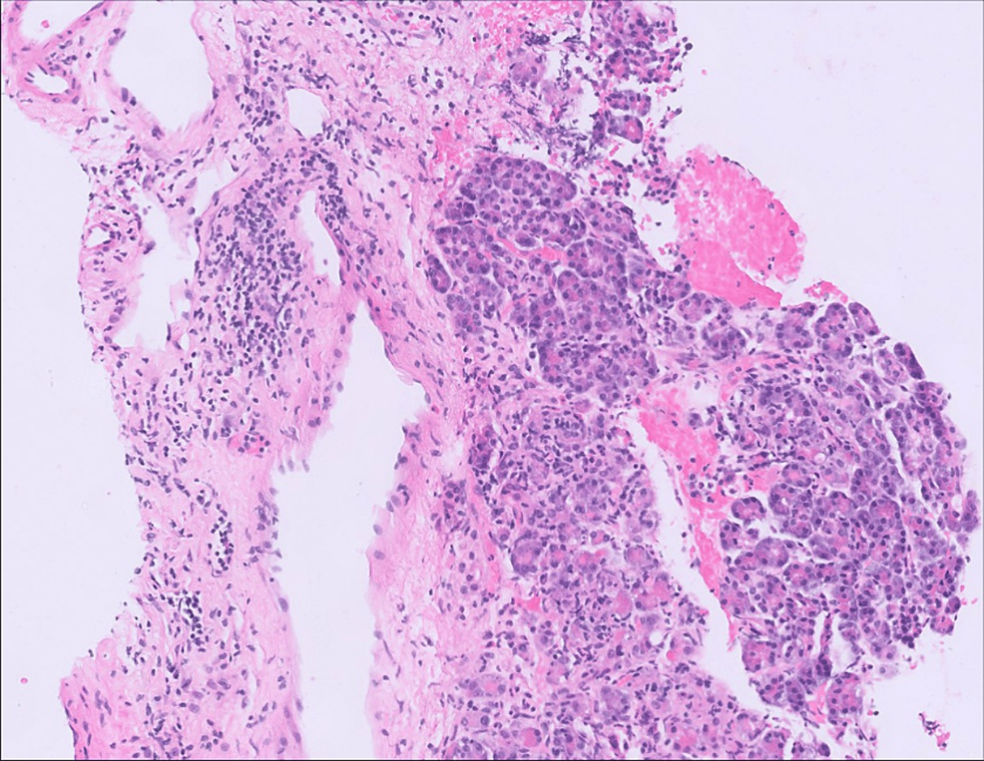
Pancreatic parenchyma with acute cellular rejection, grade II, with septal inflammation as activated lymphocytes involving septal structures and venulitis (subendothelial accumulation of inflammatory cells). Also shown is multifocal (but not confluent or diffuse) acinar inflammation (≥3 foci per lobule) with spotty acinar cell injury (power of field: 100x)

A 42-year-old male with a history of chronic hypertension, diabetes mellitus (type II), kidney transplant in 2002 due to end-stage renal disease, and a subsequent pancreas-after-kidney (PAK) transplant in 2020 due to diabetes mellitus, presented with five days of myalgias, subjective fevers, and lower extremity nodules. The patient was afebrile and hemodynamically stable. Erythematous and tender subcutaneous nodules were noted on the lower extremities (Figure 1). Two weeks prior, the patient was referred for hospital admission following undetectable tacrolimus levels and elevated lipase and amylase levels at 708 U/L (reference <60 U/L) and 595 U/L (reference <100 U/L), respectively. Further evaluation demonstrated biopsy-proven acute pancreatic allograft rejection and baseline kidney function. The pancreatic allograft biopsy revealed septal inflammation with activated lymphocytes involving septal structures and venulitis with subendothelial accumulation of inflammatory cells. Additionally, multifocal acinar inflammation is present in at least three foci per lobule, accompanied by spotty acinar cell injury. Both observations were made at a 100x power of field (Figure 2).

**Figure 3:**
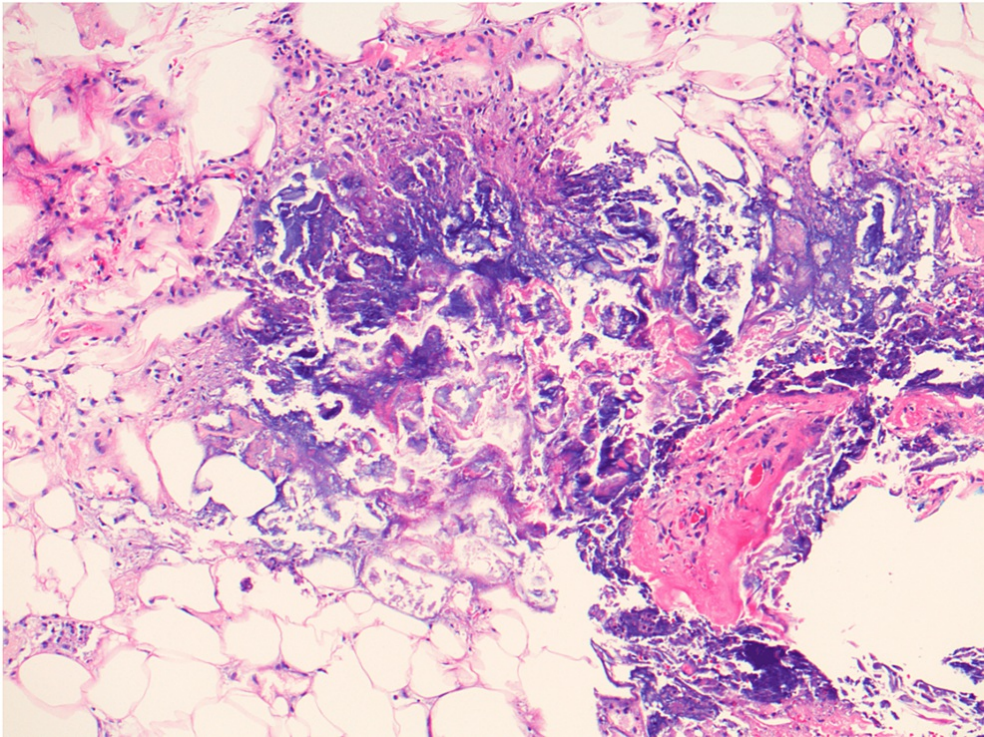
Hematoxylin and Eosin stain (100x) demonstrating ghosts of lipocytes surrounded by basophilic material and a mixed inflammatory cell infiltrate, consistent with pancreatic panniculitis

A punch biopsy of the right lower extremity demonstrated lobular panniculitis with necrotic adipocytes, basophilic debris, and calcification, consistent with PP (Figures 1, 3). Notable laboratory values included serum lipase and amylase elevations to 1781 U/L and 881 U/L, respectively. Kidney function remained at baseline. The patient's clinical history, elevated pancreatic enzymes, and histopathology supported a diagnosis of PP in the setting of acute pancreas transplant rejection. Pancreatic rejection was treated with pulse methylprednisolone and thymoglobulin. Once pancreatic enzyme levels trended down, the dosage of prednisone, tacrolimus, and mycophenolate were increased. Conservative therapy improved his symptoms and resolved the nodules (pain control, leg elevation, and topical triamcinolone).

## Discussion

PP is a rare entity, frequently associated with pancreatitis or pancreatic cancer. Although uncommon, there have been reports of PP secondary to pancreatic transplant rejection, which is an important entity to recognize as the first clinical manifestation of rejection. To our knowledge, PP in the setting of pancreas and/or kidney allograft transplant rejection has only been reported six times (Table 1) [5-10]. Kidney allograft rejection was reported in all six cases, four of which reported concurrent pancreas allograft rejection (Table 1).

**Table 1:** Summary of pancreatic panniculitis cases in the setting of pancreatic and/or renal allograft rejection PP - pancreatic panniculitis; SPK - simultaneous pancreas-kidney; PAK - pancreas after kidney; SLE - systemic lupus erythematosus; CMV - cytomegalovirus

Author	Journal and year	Patient	Transplant	Time course until rejection	Dermatological findings	Amylase and lipase levels	Biopsy	Clinical setting	Clinical notes
Wang et al. [5]	Gastroenterology, 2000	34y M	Kidney	Kidney after three months	Nodules on lower legs and ankles	Yes	Kidney graft biopsy with acute rejection and CMV infection	Inpatient	PP and pancreatitis concurrent with kidney transplant rejection. Epigastric discomfort present.
Echeverria et al. [6]	International Journal of Dermatology, 2001	42y F	Kidney	Chronic kidney rejection	Nodules on legs and ankles	Yes	Chronic kidney graft rejection	Inpatient	PP in patient with SLE, renal transplant + pancreatitis
Pike et al. [7]	American Journal of Transplantation, 2006	49y F	SPK	Kidney and pancreas after five months	Nodules on knees and legs	Yes	Kidney with acute cellular rejection. Presumed pancreas rejection	Inpatient	PP developed 12 days after rejection pancreatitis
Prikis et al. [8]	American Journal of Transplantation, 2010	40y F	PAK	Kidney after 15 months and pancreas after 21 months	Nodules on the legs and feet	Yes	Pancreas with mixed acute cellular rejection and antibody-mediated rejection	Inpatient	PP developed days after pancreas rejection
Beveridge et al. [9]	Journal of the American Acadamy of Dermatology Case Report, 2015	34y F	SPK	Kidney - unknown time and pancreas after six years	Nodules on legs	Yes	Pancreas biopsy not performed	Outpatient	PP developed six years after SPK transplant. Further investigation prompted the finding of pancreatic rejection.
Baig et al. [10]	American Journal of Transplantation, 2019	54y M	SPK	Pancreas after four years and kidney after 13.5 years	Nodules on legs	Yes	Recent kidney rejection with chronic allograft nephropathy and chronic transplant glomerulopathy. Acute cellular rejection of kidney allograft. Pancreas biopsy not performed	Outpatient	The patient stopped taking prednisone one month prior to PP. PP developed four years after pancreas rejection and six months after the kidney rejection.
[Current Study	2024	42y M	PAK	Pancreas after 11 months	Nodules on legs, shins, and ankles	Yes	Acute pancreatic allograft rejection	Outpatient	Only case of PP to present in the setting of pancreas allograft rejection in the absence of kidney rejection and pancreatitis.

In 2000, Wang et al. described a case of PP occurring in the setting of kidney transplant failure and laboratory evidence of acute pancreatitis [5].^ ^On examination, the patient had erythematous nodules in both lower extremities. The panniculitis and laboratory abnormalities resolved after three weeks of conservative pancreatitis management and hemodialysis. This was the first case of PP associated with biopsy-proven renal allograft rejection.

In 2001, Echeverria et al. reported a case of PP occurring in the setting of renal transplant rejection [6]. This patient had systemic lupus erythematous and was hospitalized after presenting with fever, abdominal pain, vomiting, arthritis, and several bilateral lower extremity painful nodules. Histopathology of the nodules confirmed the diagnosis of PP. Imaging and laboratory evidence confirmed acute pancreatitis. Immunosuppressive therapy was stopped, and conservative management resulted in full resolution of skin nodules. This case, where the skin nodules presented in parallel with abdominal and epigastric symptoms, was instructive as it demonstrated that halting immunosuppressive therapy resolved the panniculitis lesions. 

In 2006, Pike et al. reported PP occurring in a patient with diabetes and chronic renal failure who had previously undergone a simultaneous pancreas-kidney (SPK) transplant [7]. Four months after the transplant, the patient experienced elevated creatinine levels, abdominal pain, and diarrhea that resolved after a two-day hospitalization. One month later, the patient developed increased creatinine, lipase, and amylase levels and was admitted for closer evaluation. Biopsy results confirmed both pancreas and kidney allograft rejection. On hospital days 8-12, several tender and erythematous nodules developed on both legs, and dermatopathology results confirmed PP. Treatment of this patient's renal allograft and pancreas rejection resolved the PP.

In 2010, Prikis et al. reported the first case of PP occurring in a patient with a pancreas after a kidney (PAK) transplant and secondary antibody-mediated pancreas allograft rejection [8]. The patient had elevated donor-specific antibodies (DSA) against both kidney and pancreas donor organs and an elevated creatinine up to 2.2mg/dL (baseline 1.5-1.9mg/dL). This finding is in direct contrast to our case, where there was no evidence of kidney damage or rejection. The serum amylase and lipase were also both elevated to 1063 U/L and 1331 U/L, respectively.

In 2015, Beveridge et al. reported a case of PP in the setting of pancreas and kidney allograft rejection. The rejections occurred six years after organ transplantation [9]. Even without fever, abdominal pain, or systemic symptoms, the patient developed nodules on the anterior shins. Histopathology confirmed PP. Clinical tests indicated allograft rejection and acute pancreatitis. Elevated amylase, lipase, and undetectable cyclosporine levels supported pancreas allograft rejection, even without a biopsy. Modifying the immunosuppressive regimen resolved the panniculitis and other abnormalities.

In 2019, Baig et al. reported a case of PP occurring 14 years after SPK transplant [10]. The patient developed pancreas rejection and renal allograft failure four years and 14 years after the SKP transplant, respectively. Due to this rejection, chronic immunosuppressive medications were stopped, and dialysis was started. A month later, the patient had fevers and developed bilateral nodules on the lower extremities. Biopsy confirmed PP, and other tests indicated acute allograft pancreatitis. Initiation of systemic steroids and immunosuppressive therapy resolved the nodules.

Our case report of PP is unique in two notable ways. Firstly, it is the second reported case to occur secondary to acute pancreas allograft rejection in a PAK recipient. Secondly, it is the only known case of PP to occur in the setting of pancreatic allograft rejection without evidence of simultaneous kidney rejection. The patient's creatinine and eGFR values at the initial evaluation were 1.47 mg/dL and 58 mL/min/BSA, respectively, falling within the normal baseline range for the patient. Notably, this patient's kidney function remained at baseline prior to, during, and after the resolution of both his pancreas allograft rejection and PP. Our case report presents a rare occurrence of PP that developed because of isolated pancreas allograft rejection in a PAK recipient, highlighting key clinical features and patient characteristics relevant to this presentation in transplant patients. Unlike previously reported case studies of PP in transplant recipients, our case did not involve kidney transplant rejection or dysfunction. Thus, it provides valuable guidance for clinicians in diagnosing and managing patients who show clinical evidence of PP in the presence of isolated pancreas allograft rejection and normal kidney function. Initial signs of this condition can manifest as tender, erythematous, and violaceous nodules on the lower extremities and trunk. In the context of a pancreas or kidney transplant recipient, allograft rejection should be strongly considered, and a prompt clinical workup should be performed.

## Conclusions

We present a unique case of pancreatic panniculitis (PP) secondary to acute pancreas allograft rejection in a PAK transplant patient. Although PP is commonly associated with pancreatitis, pancreatic cancer, or native pancreatitis in pancreas and/or kidney transplant recipients, it is uncommonly reported to occur in the setting of pancreas transplant rejection without simultaneous kidney damage or rejection. A prompt diagnosis of PP and management of the underlying cause, in this case, acute pancreas allograft rejection, is essential to achieve a favorable clinical outcome. Open and collaborative communication across physician specialties is essential, as the treatment of PP skin manifestations is directed at managing the underlying pancreatic pathology. Lastly, this case emphasizes the importance of screening for underlying pancreatic disease in patients with PP.
